# Inhibitory role of bone marrow mesenchymal stem cells‐derived exosome in non‐small‐cell lung cancer: microRNA‐30b‐5p, EZH2 and PI3K/AKT pathway

**DOI:** 10.1111/jcmm.17933

**Published:** 2023-09-12

**Authors:** Tong Wu, Qi Tian, Ruiji Liu, Ke Xu, Shanshan Shi, Xiudi Zhang, Liming Gao, Xiaobo Yin, Shufeng Xu, Ping Wang

**Affiliations:** ^1^ Graduate School of Zunyi Medical University Zunyi China; ^2^ Department of Pulmonary and Critical Care Medicine The First Hospital of Qinhuangdao Qinhuangdao China; ^3^ Graduate School of Hebei Medical University Shijiazhuang China; ^4^ Oncology Department The First Hospital of Qinhuangdao Qinhuangdao China; ^5^ Department of Pulmonary and Critical Care Medicine Chinese People's Liberation Army General Hospital Beijing China

**Keywords:** bone marrow mesenchymal stem cells, exosome, EZH2, microRNA‐30b‐5p, non‐small‐cell lung cancer, PI3K/AKT

## Abstract

Exosomal microRNA (miRNA) exerts potential roles in non‐small‐cell lung cancer (NSCLC). The current study elucidated the role of miR‐30b‐5p shuttled by bone marrow mesenchymal stem cells (BMSCs)‐derived exosomes in treating NSCLC. Bioinformatics analysis was performed with NSCLC‐related miRNA microarray GSE169587 and mRNA data GSE74706 obtained for collection of the differentially expressed miRNAs and mRNAs. The relationship between miR‐30b‐5p and EZH2 was predicted and confirmed. Exosomes were isolated from BMSCs and identified. BMSCs‐derived exosomes overexpressing miR‐30b‐5p were used to establish subcutaneous tumorigenesis models to study the effects of miR‐30b‐5p, EZH2 and PI3K/AKT signalling pathway on tumour growth. A total of 86 BMSC‐exo‐miRNAs were differentially expressed in NSCLC. Bioinfomatics analysis found that BMSC‐exo‐miR‐30b‐5p could regulate NSCLC progression by targeting EZH2, which was verified by in vitro cell experiments. Besides, the target genes of miR‐30b‐5p were enriched in PI3K/AKT signalling pathway. Animal experiments validated that BMSC‐exo‐miR‐30b‐5p promoted NSCLC cell apoptosis and prevented tumorigenesis in nude mice via EZH2/PI3K/AKT axis. Collectively, the inhibitory role of BMSC‐derived exosomes‐loaded miR‐30b‐5p in NSCLC was achieved through blocking the EZH2/PI3K/AKT axis.

## 1 INTRODUCTION

Lung cancer remains the leading cause of cancer‐related death around the world with non‐small‐cell lung cancer (NSCLC) accounts for ~85%.^1^, ^2^ There are multiple risk factors for the progression of NSCLC, including cooking oil vapour, smoke and genetic susceptibility.^3^ Although there are great advancements in the treatment of NSCLC, including targeted therapy and immunotherapy, the prognosis of patients with NSCLC remains poor.^4^, ^5^

Bone marrow‐derived mesenchymal stem cells (BMSCs), pluripotent stromal cells that are recruited into tumours, are involved in the development of several malignant tumour, including NSCLC.^6^ BMSCs can release exosomes to participate in tumorigenesis, acting as a potential therapeutic strategy for cancer patients with NSCLC.^7^ Exosomes, ranging from 30 to 150 nm in diameter, could deliver various biomolecules, including mRNAs, microRNAs (miRNAs) and lipids to affect the tumour progression.^8^, ^9^ Specifically, BMSC‐derived exosomes carrying miRNAs are implicated in the metastasis of NSCLC.^6^, ^10^ One recent has highlighted the inhibitory role of miR‐30b‐5p in lung cancer,^11^ while its role in NSCLC remains to be elucidated.

Interestingly, enhancer of zeste homologue 2 (EZH2) was predicted to be one of the target genes of miR‐30b‐5p by TargetScan and miRWalk database in the present study. EZH2 is the catalytic subunit of PRC2 that modulates suppression of target genes through trimethylation of lysine 27 on histone H3.^12^ EZH2 acts as a main modulator of cell autophagy and apoptosis to affect progression of various cancers.^13^ Specifically, the critical role of EZH2 in NSCLC has also been highlighted.^14^, ^15^ More importantly, a previous study has elicited that UFC1 overexpression elevates EZH2 to activate PI3K/Akt pathway, thus facilitating the tumorigenesis of NSCLC.^16^ It is interesting to note that inhibition of PI3K/AKT signalling pathway suppresses the NSCLC progression.^17^ However, the roles of BMSC‐derived exosomes‐mediated miR‐30b‐5p in NSCLC have not been elaborated. In our current study, we combined in silico analysis and in vitro and in vivo assays to identify the critical function of BMSC‐derived exosomes‐mediated miR‐30b‐5p in NSCLC.

## 2 MATERIALS AND METHODS

### 2.1 Data sources

NSCLC‐related miRNA data GSE169587^18^ and mRNA data GSE74706^19^ were downloaded from the Gene Expression Omnibus (GEO) database. GSE169587 contained 7 NSCLC tissue samples and 12 adjacent normal tissue samples, and GSE74706 included 18 NSCLC tissue samples and 18 adjacent normal tissue samples. The dataset was based on the platform information GPL25134 and GPL13497 to annotate the gene ID on the microarray dataset.

### 2.2 Weighted gene co‐expression network analysis

Weighted gene co‐expression network analysis (WGCNA) analysis^20^ was performed using the R software ‘WGCNA’ package. First, Hclust function was used to conduct hierarchical clustering analysis, and the appropriate soft threshold β was selected using the ‘pickSoftThreshold’ function, followed by adjacency matrix transformation. The topological overlap matrix (TOM) was calculated, and the hierarchical cluster tree diagram was constructed. The similar gene expression was divided into different modules with 50 as the minimum number of genes in the module. In order to merge possible similar modules, 0.25 was defined as the threshold of cutting height. Finally, the expression profile of each module was summarized by the module eigengene (ME), and the correlation between the ME and the traits was calculated.

### 2.3 Differential gene expression analysis

The differentially expressed miRNAs or differentially expressed genes (DEGs) in the GSE169587 and GSE74706 were obtained using the R language ‘limma’ package with |logFC| > 1 and *p* < 0. 05. Volcano maps were drawn using the R language ‘ggplot2’ package, and the heat maps of DEGs were drawn using the R software ‘heatmap’ package. BMSC‐exo‐miRNAs were screened out using the EVmiRNA database, and the Venn map was obtained using the Online analysis tool Xiantao Academic. The TargetScan and miRWalk databases were used to predict the target genes of miRNA.

### 2.4 Functional enrichment analysis

GO and pathway enrichment analysis of differential miRNA was performed to identify the biological functions (including biological processes [BPs], molecular functions [MFs] and cellular components [CCs]) and pathway using FunRichR software. An online database was utilized for GO (including BPs, MFs and CCs) and KEGG pathway enrichment analysis (www.kegg.jp/kegg/kegg1.html) on the differential mRNAs.

### 2.5 Expression of the target genes in the microarray data

The expression of miR‐30b‐5p and EZH2 in the NSCLC‐related miRNA data (GSE169587) and mRNA data (GSE74706) was extracted, respectively.

### 2.6 Cell culture and transduction

Human BMSCs (CP‐H166, Procell Life Science & Technology Co., Ltd.) were cultured in BMSC complete medium (CM‐H166, Procell Life Science & Technology). Human kidney cells HEK‐293T (CL‐0005, Procell Life Science & Technology) were incubated in Dulbecco's modified Eagle's medium (DMEM; PM150210, Procell Life Science & Technology) supplemented with 10% foetal bovine serum (FBS, 164210, Procell Life Science & Technology), 100 U/mL penicillin and 100 U/mL streptomycin (PB180120, Procell Life Science & Technology). Human NSCLC cell A549 cells (CL‐0016, Procell Life Science & Technology) and NCI‐H1299 cells (CL‐0165, Procell Life Science & Technology) were cultured in Ham's F‐12K medium (PM150910, Procell Life Science & Technology) containing 10% FBS (164,210, Procell Life Science & Technology), 100 U/mL penicillin and 100 U/mL streptomycin (PB180120, Procell Life Science & Technology), which were placed in an incubator with 5% CO_2_ at 37°C.

Lentivirus packaging system was constructed through the LV5‐GFP (overexpression vector of lentivirus gene) and pSIH1‐H1‐copGFP (silencing vector of lentivirus gene). The packaging lentivirus and the target vector were co‐transduced into 293T cells with lipofectamine 2000 (80%–90% of cell confluence). After incubation for 48 h, the supernatant was collected. The supernatant containing lentivirus particles after centrifugation was filtered. The lentivirus in the exponential growth phase was used to detect the virus titre. Cells in logarithmic growth phase were detached with trypsin and triturated to prepare cell suspension (5 × 10^4^ cells/mL), which was seeded into the 6‐well plates (2 mL/well). Next, the lentivirus with titre of 1 × 10^9^ TU/mL was added into the plate with cell multiplicity of infection (MOI) of 10. After 48 h, cells were collected for further experiments. A549 cells and NCI‐H1299 cells were transduced with negative control (NC) mimic (5′‐UACUGAGAGACAUAAGUUGGUC‐3′), miR‐30b‐5p mimic (5′‐UGUAAACAUCCUACACUCAGCU‐3′), NC inhibitor (5′‐CAGUACUUUUGUGUAGUACAA‐3′), miR‐30b‐5p inhibitor (5′‐AGAACAGUGAAAUUUCCAGUCC‐3′), overexpression (oe)‐NC, or oe‐EZH2, while BMSCs were transduced with NC mimic and miR‐30b‐5p mimic, respectively. Lentiviruses were purchased from Shanghai GenePharma Co. Ltd. After transduction for 48 h, cells were collected for mRNA and protein extraction, followed by reverse transcription‐quantitative polymerase chain reaction (RT‐qPCR) and Western blot analysis.

### 2.7 RNA extraction and quantification

Total RNA was extracted from tissues and EVs using the TRIZOL (16,096,020, Thermo Fisher Scientific). miRNA detection was conducted using the PolyA tailing test kit (including Universal PCR primer R and U6 Universal PCR primer R) (B532451, Shanghai Sangon Biotechnology Co. Ltd.) to obtain the cDNA of miRNA containing PolyA tail. RT of mRNA was performed using the cDNA reverse transcription kit (RR047A, Takara). PCR was completed utilizing LightCycler 480 SYBR Green I Master (04707516001, Roche). U6 served as an internal reference for miRNA and glyceraldehyde‐3‐phosphate dehydrogenase (GAPDH) as an internal reference for other genes. The primer sequences are shown in Table S1. The $2^{-\Delta \Delta C_{t}}$ method was used to quantify relative expression levels of target genes.

### 2.8 Western blot analysis

The tissues, Exo and cells were lysed by Radio Immunoprecipitation Assay containing phenylmethylsulfonyl fluoride (P0013B, Beyotime Institute of Biotechnology) to extract total protein, followed by determination of protein concentration using the bicinchoninic acid kit (P0028, Beyotime). The protein was separated by electrophoresis and then transferred to a polyvinylidene fluoride membrane (1,620,177, Bio‐Rad), which was sealed with 5% bovine serum albumin at room temperature for 1 h. Next, the protein was incubated with diluted primary antibody EZH2 (ab186006,1:1000, Rabbit, Abcam) at 4°C overnight and with secondary antibody horseradish peroxidase (HRP)‐labelled goat anti‐rabbit against immunoglobulin G (IgG) (ab6721, 1: 5000, Abcam) at room temperature for 1 h. After washing, the membrane was immersed in enhanced chemiluminescence, followed by imaging on an Image Quant LAS 4000C gel imager. GAPDH (A01021, 1: 5000, Rabbit, abbkine) served as the internal reference. The grey value of protein bands was quantified using Image J analysis software with Tubulin as the internal reference.

### 2.9 Dual‐luciferase report gene assay

The EZH2 3′‐UTR wild type (WT) or mutant (MUT) sequences were synthetized, which were cloned into the pMIR‐reporter utilizing luciferase report gene kit (Promega Corporation). The EZH2 3′‐UTR WT or MUT sequences were co‐transduced into HEK293T cells with miR‐30‐5p mimic or NC mimic using the Lipofectamine 2000 reagent kit. After incubation for 48 h, cells were collected and lysed. The luciferase activity was measured using the Promega luciferase detection kit and dual Glomax 20/20 luminometer fluorescence detector (Promega).

### 2.10 Directional differentiation and identification of BMSCs

The isolated BMSCs were cultured with DMEM adipogenic induction medium containing 10^7^ mol/L dexamethasone (HY‐14648, MedChemExpress [MCE]), 5 mg/L insulin (HY‐P0035, MCE), 0.5 mmol/L 3‐isobutyl xanthine (HY‐12318, MCE), 60 μmol/L inddomexine (HY‐14397, MCE) and 10% FBS for 14‐day of induction to obtain adipocytes. The isolated BMSCs were cultured with DMEM osteogenic induction medium containing 10^7^ mol/L dexamethasone (HY‐14648, MCE), 50 μg/L vitamin C (HY‐103701, MCE), 2 mmol/L β‐glycerophosphate (HY‐D0886, MCE) and 10% FBS for 14‐day of induction to obtain osteoblasts. The isolated BMSCs were cultured with DMEM high sugar solution containing 100 ng/LIL‐5 (HY‐P7043, MCE), 10^7^ mol/L dexamethasone, 50 μg/L vitamin C (HY‐103701, MCE), 1% FBS, 1% ITS+ (I3146‐5ML, Sigma‐Aldrich), 40 μg/mL L‐Proline (HY‐Y0252, MCE) and 100 μg/mL sodium pyruvate (HY‐W015913, MCE) for 14 days of induction to obtain chondrocytes. All the above‐mentioned culture medium was changed every 3 days. Alizarin red staining (ST1078, Beyotime) was employed to detect osteogenic differentiation ability, Oil red O staining (E607319, Sangon Biotech) to detect adipogenic differentiation ability, and Alcian Blue staining (A600298, Sangon Biotech) to detect chondrogenic differentiation ability of BMSCs. The BMSC surface markers CD44 (ab243894, Abcam), CD73 (ab257309, Abcam), CD90 (ab23894, Abcam) and CD34 (ab81289, Abcam) were detected by flow cytometry.

### 2.11 Isolation and identification of exosomes

Exosomes (exo‐NC mimic and exo‐miR‐30b‐5p mimic) were isolated from the culture medium supernatants of BMSCs transduced with NC mimic or miR‐30b‐5p mimic using the differential centrifugation.^21^ Exosomes were cultured in the medium with Exo‐free serum (EXO‐FBS‐50A‐1, Systembio, Shanghai, China).

Exosomes were identified with a transmission electron microscope (TEM; H‐7650, HITACHI, Tokyo, Japan). The exosomes surface‐labelled proteins were identified by Western blot analysis with the antibodies as follows: CD63 (ab271286, 1:1000, Mouse, Abcam), CD81 (ab79559, 1:1000, Mouse, Abcam), TSG101 (ab125011, 1:2000, Rabbit, Abcam) and calnexin (ab133615, 1:2000, Rabbit, Abcam). The sizes of exosomes size were analysed by Nanoparticle Tracking Analysis (NTA) using the nanoparticle tracking analyser (NanoSightLM10, Malvern Panalytical, UK).

### 2.12 Exosome uptake assay

BMSC exosomes were labelled by PKH67 green fluorescence according to the manufacturer's instructions (PKH26GL, Sigma‐Aldrich), followed by incubation at room temperature for 15 min and centrifugation at 1000 *g* for 5 min to remove the supernatant. The BMSC exosomes were resuspended and centrifuged at 1000 *g* for 5 min to remove the supernatant. The precipitate obtained after two repetition was PKH26‐labelled BMSC exosomes. The cell specific slides were placed on the top of the culture dish. A549 cells or NCI‐H1299 cells were placed into the culture dish. Upon cell density reached 50%, the cells were added with PKH26‐labelled BMSC exosomes at 37°C for 24 h. Cell slides were taken out and washed with PBS three times. The cells were immersed in 4% paraformaldehyde, washed in PBS three times, permeabilized with 2% Triton X‐100 for 15 min and stained with 4′, 6‐diamino‐2‐phenylindole (2 μg/mL, C1005, Beyotime) for 10 min. After that, the expression of the fluorescence was observed under the confocal laser scanning microscope.

### 2.13 Animal experiments

BALB/c male nude mice were purchased from Beijing Charles River Laboratory Animal Technology Ltd., and mice were raised in the laboratory of specific pathogen free at 22–25°C with humidity of 60%–65%. The experiment was conducted after acclimatization for 1 week. The animal protocol was approved by animal ethics committee of Chinese People's Liberation Army General Hospital.

A549 cells transduced with oe‐NC and oe‐EZH2 were subcutaneously injected into the right armpit of nude mice (1 × 10^6^ cells/mice). When the tumour volume reached 100 mm^3^, nude mice were injected with exo‐NC and exo‐miR‐30b‐5p (200 μg/mice), once every 2 days for a total of 10 times. The AKT agonist SC79 (10 mg/kg, H Y‐18749, MCE) was injected around the tumour body at 0 h before exosomes injection. After 4 weeks, tumour volume was measured and weighed, volume = (length × width^2^)/2. All mice were injected with oe‐NC, oe‐EZH2, oe‐EZH2 + exo‐NC, oe‐EZH2 + exo‐miR‐30b‐5p and oe‐EZH2 + exo‐miR‐30b‐5p + SC79.

### 2.14 Immunohistochemistry

The paraffin‐embedded sections of NSCLC tissues were dehydrated and subjected to antigen retrieval in water bath at 100°C. After cooling down with running water, the tissues were added with 5% goat serum blocking solution (C0265, Beyotime) and proved with primary antibody EZH2 (ab186006, 1:250, Rabbit, Abcam), PTEN (ab267787, 1:200, Abcam), p‐AKT (#4060, 1:200, Rabbit, CST), AKT (#4685, 1: 200, Rabbit, CST), p‐PI3K (ABP0163, 1: 200, Rabbit, Abbkine), PI3K (ABP50495, 1: 200, Rabbit, Abbkine) and Ki‐67 (ab15580, 1: 100, Rabbit, Abcam) at 4°C overnight. Next, the tissues were added with the secondary antibody goat anti‐rabbit IgG (ab6721, 1:1000, Abcam) at 37°C for 20 min and HRP‐labelled Streptavidin (A0303, Beyotime) at 37°C for 20 min. Subsequently, the tissues were developed with DAB (P0203, Beyotime), stained with haematoxylin (C0107, Beyotime) for 1 min, washed, treated with 1% ammonia for back to blue, dehydrated with gradient alcohol of a certain concentration, cleared by xylene, sealed with neutral resin, and observed and captured under the microscope (BX63, Olympus). The images were reviewed by an experienced pathologist; five visual fields were randomly selected and analysed, 100 cells per field. The percentage of positively stained cells was calculated. Immunohistochemistry (IHC) score = A × B (A: 0: no positively stained cells, 1: 10% positive cells, 2: 11%–50% positive cells, 3: 51%–80% positive cells, 4: more than 80% positive cells; B: 0: no staining, 1: shallow staining intensity, 2: medium staining intensity, 3: deep staining intensity).

### 2.15 TUNEL staining

Paraffin‐embedded sections of NSCLC tissues were fixed in 4% paraformaldehyde for 15 min, washed in PBS for 3 times (5 min for each time) and treated with 20 g/mL Proteinase K for 15 min. After that, the sections were incubated in endogenous peroxidase blocking solution (P0100A, Beyotime) for 20 min at room temperature to inactivate the endogenous peroxidase. Subsequently, the sections were stained with TUNEL staining kit (C1091, Beyotime), incubated with 50 μL biotin labelling solution at 37°C for 60 min and incubated with 0. 3 mL labelled reaction termination solution for 10 min at room temperature. After incubation with 50 μL Streptavidin‐HRP working solution for 30, the sections were incubated with 0.5 mL DAB colour development solution for 5 min at room temperature. The images were observed under the microscope, and the brown cells were apoptotic cells. The proportion of apoptotic cells in each group was calculated by Image pro plus 6.0 software.

### 2.16 Statistical analysis

Data analysis was performed using the R software v4.1.1 (R Foundation for Statistical Computing) and SPSS 21.0 software (IBM). Spearman correlation analysis was used to detect the correlation among the observed indicators. All quantitative data are presented as mean ± standard deviation. The normality and homogeneity of variance were conducted. The data conforming to normal distribution and homogeneous variance between two groups were analysed by unpaired *t*‐test, followed by Tukey's post hoc test. Data comparisons among multiple groups were analysed by the one‐way anova with Tukey's post hoc test, and data comparisons at different time points were analysed by the two‐way anova or repeated measures anova with Bonferroni post hoc test. *p* < 0.05 was considered as statistically significant.

## 3 RESULTS

### 3.1 Differential gene analysis selected 86 differentially expressed BMSC‐exo‐miRNAs in NSCLC

**FIGURE 1:**
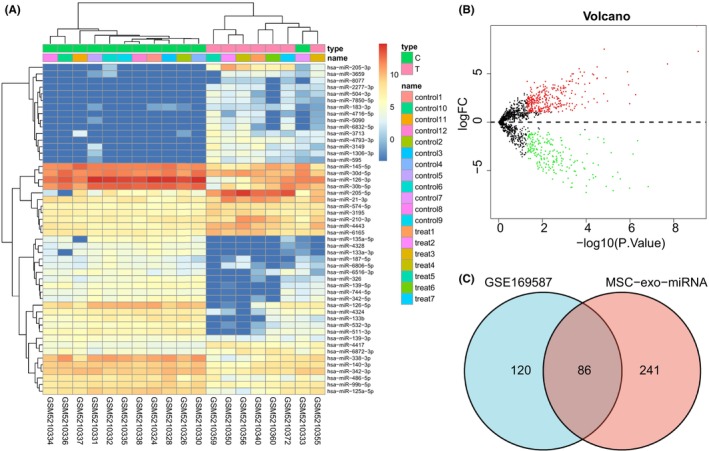
Screening and functional enrichment analysis of differentially expressed miRNAs in microarray data GSE169587. (A) Heat map of differentially expressed miRNAs in the normal control group (*n* = 12) and NSCLC group (*n* = 7) in microarray data GSE169587. Green indicates the downregulated miRNAs; red indicates the upregulated miRNAs; and black indicates miRNAs without significant difference. (B) Volcano map of differentially expressed miRNAs in microarray data GSE169587. Green dots indicate the downregulated miRNAs; red dots indicate the upregulated miRNAs; and black dots indicate miRNAs without significant difference. (C) Venn diagram of differentially downregulated miRNAs in BMSC‐exo‐mRNA and GSE169587.

BMSC‐derived exosomes have been shown to play essential roles in NSCLC.^7^ At first, the NSCLC‐related miRNA microarray data GSE169587 were retrieved from the GEO database, followed by the differential analysis to select the miRNAs with significant differential expression between the normal control and NSCLC groups. Finally, 517 differentially expressed miRNAs were selected, of which 206 were downregulated and 311 were upregulated (Figure 1A,B; Table S2). Moreover, there were 327 BMSC‐exo‐miRNAs retrieved from the EVmiRNA database, which were intersected with the downregulated differentially expressed miRNAs obtained from GSE169587. Finally, 86 differentially expressed BMSC‐exo‐miRNAs were obtained (Figure 1C; Table S3).

Next, the GO and KEGG analysis of the 86 differentially expressed BMSC‐exo‐miRNA were performed using the FunRich software. The results of GO function analysis showed that in CC, the differentially expressed miRNAs were mainly enriched in cytoplasm, nucleus, plasma membrane, golgi apparatus and lysosome. In MF, the differentially expressed miRNAs were mainly enriched in transcription factor activity, transcription regulator activity, ubiquitin‐specific protease activity and cell adhesion molecule activity. In BP, the differentially expressed miRNAs were mainly enriched in regulation of nucleobase/nucleoside/nucleotide/nucleic acid metabolism, signal transduction and cell communication (Figure S1A–C), suggesting that these miRNAs might be involved in intercellular information exchange. The results of KEGG pathway analysis exhibited that the differentially expressed miRNAs were mainly enriched in TRAIL signalling pathway and VEGF and VEGFR signalling network (Figure S1D).

### 3.2 BMSC‐exo‐miRNAs may be involved in NSCLC progression by targeting EZH2

**FIGURE 2:**
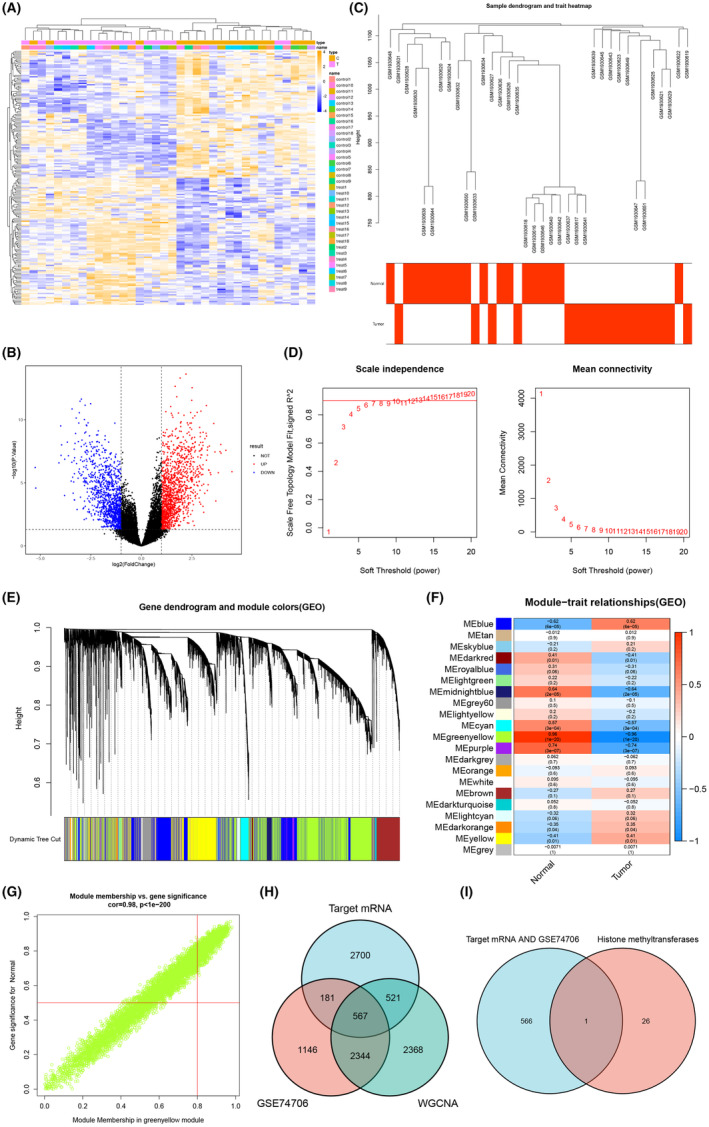
Screening and functional analysis of DEGs in microarray data GSE74706. (A) Heat map of differentially expressed mRNA in normal control (*n* = 18) and NSCLC (*n* = 18) tissues in microarray data GSE74706. (B) Volcano map of differentially expressed mRNAs in microarray data GSE74706. (C) Clustering tree of 36 samples (Below is the location of sample). (D) The scale‐free fitting index (left) and average connectivity (right) of various soft threshold power β. (E) Cluster tree of co‐expressed genes (top). (F) Heat map of the correlation of modules and traits in NSCLC. G, Scatter plot of gene significance (GS) and module members (MM) in the green yellow module. (H) Venn diagram of intersection of top 10 differentially expressed BMSC‐exo‐miRNA target genes and differential mRNAs screened from GEO microarray data by WGCNA analysis and differential analysis. (I) Venn map of the intersection between histone methyltransferases and 567 intersected target genes.

NSCLC‐related mRNA microarray data GSE74706 were retrieved from GEO database, followed by differential analysis to select the mRNAs with significant expression difference between normal control and NSCLC groups. Finally, 4238 differentially expressed mRNAs were selected, of which 2381 differential mRNAs were downregulated and 1857 differential mRNAs were upregulated (Figure 2A,B; Table S4).

Next, WGCNA analysis was performed on the microarray data GSE74706 (Figure 2C), followed by hierarchical clustering of the samples with *β* = 6 as soft threshold to establish a scale‐free network (Figure 2D). A total of 21 co‐expression modules were identified in the NSCLC expression spectrum, with each colour representing a different module. The module feature correlation analysis showed that the greenyellow module had the highest absolute value of correlation, including 5800 genes (Figure 2E), suggesting that the genes contained in the greenyellow module were the one most correlated with NSCLC (Figure 2F,G).

The target gene analysis of top 10 differentially expressed BMSC‐exo‐miRNAs (hsa‐miR‐30b‐5p, hsa‐miR‐218‐5p, hsa‐miR‐133a‐3p, hsa‐miR‐335‐5p, hsa‐miR‐532‐3p, hsa‐miR‐150‐5p, hsa‐miR‐126‐3p, hsa‐miR‐140‐5p, hsa‐miR‐582‐5p and hsa‐miR‐98‐5p) was conducted through Targetscan, miRWalk and mirDIP databases. A total of 3970 target genes were obtained, which were intersected with the differentially expressed mRNA through the WGCNA analysis and differential analysis in GEO microarray data, yielding 567 intersected target genes (Figure 2H; Table S5).

Through literature review, we screened out 27 common histone methyltransferases,^22^ which were intersected with 567 intersected target genes, and finally the intersected gene EZH2 was obtained (Figure 2I).

It could be speculated that BMSC‐exo‐miRNAs may participate in NSCLC progression by targeting EZH2.

### 3.3 miR‐30b‐5p could target and inhibit EZH2 in NSCLC

**FIGURE 3:**
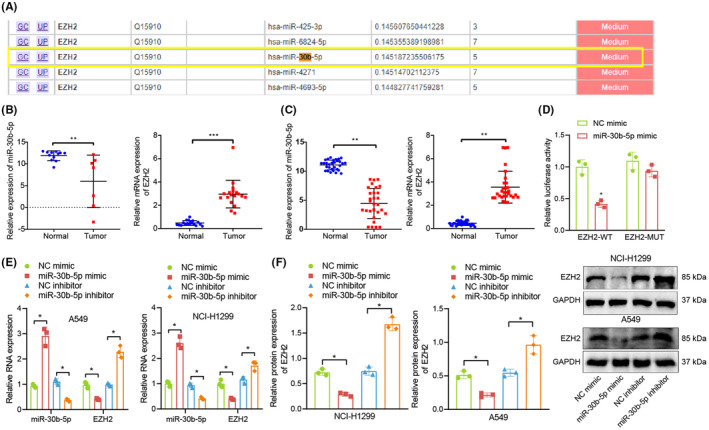
Validation of the target relationship between miR‐30b‐5p and EZH2. (A) The targeted binding site between hsa‐miR‐30b‐5p and EZH2 predicted in the mirDIP database. (B, C) Expression of miR‐30b‐5p and EZH2 in GSE169587 (normal control group: *n* = 12, NSCLC group: *n* = 7) and GSE74706 (normal control group: *n* = 18, NSCLC group: *n* = 18), respectively. (D) Target relationship between miR‐30b‐5p and EZH2 detected by dual‐luciferase reporter gene assay. (E, F) EZH2 expression in A549 cells or NCI‐H1299 cells transduced with miR‐30b‐5p mimic or miR‐30b‐5p inhibitor determined by RT‐qPCR and Western blot analysis. *p < 0.05, **p < 0.01 and ***p < 0.001.

mirDIP database predicted that there were targeted binding sites between hsa‐miR‐30b‐5p and EZH2 (Figure 3A). Therefore, we speculated that BMSC‐exo‐miR‐30b‐5p may participate in the development of NSCLC by targeting EZH2.

To test this speculation, we extracted the expression of miR‐30b‐5p and EZH2 in GSE169587 and GSE74706, respectively, which exhibited that miR‐30b‐5p was downregulated in NSCLC, and EZH2 was highly expressed in NSCLC (Figure 3B,C).

Dual‐luciferase reporter gene assay exhibited that luciferase activity of EZH2‐WT was obviously inhibited by miR‐30b‐5p mimic, while EZH2‐MUT showed no obvious difference, suggesting that miR‐30b‐5p targeted and negatively regulated EZH2 (Figure 3D).

The results of RT‐qPCR and Western blot analyses showed that overexpression of miR‐30b‐5p significantly increased the expression levels of miR‐30b‐5p in A549 and/or NCI‐H1299 cells, while concomitantly decreasing the levels of EZH2 mRNA and protein. On the contrary, inhibition of miR‐30b‐5p expression led to a significant decrease in the expression levels of miR‐30b‐5p in A549 and/or NCI‐H1299 cells, while concomitantly increasing the levels of EZH2 mRNA and protein (Figure 3E,F).

These results indicated that miR‐30b‐5p could target and inhibit EZH2 expression in NSCLC.

### 3.4 PI3K/AKT signalling pathway is involved in the occurrence and development of NSCLC based on functional enrichment analysis

Next, the GO function and KEGG pathway enrichment analysis were performed on the target genes of miR‐30b‐5p. The results of GO functional analysis represented that in BP, the target genes were mainly enriched in ameboidal‐type cell migration, embryonic organ development and urogenital system development. In CC, the target genes were mainly enriched in cell–cell junction, synaptic membrane and neuron to neuron synapse. In MF, the target genes were mainly enriched in glycosaminoglycan binding, growth factor binding and sodium ion transmembrane transporter activity (Figure S2A). KEGG pathway displayed that the target genes were mainly enriched in Wnt signalling pathway, PI3K‐AKT signalling pathway and MAPK signalling pathway (Figure S2B). Thus, we speculated that miR‐30b‐5p could inhibit EZH2 to regulate the activation of PI3K/AKT signalling pathway, thus participating in the occurrence and development of NSCLC.

### 3.5 BMSC‐exo‐miR‐30b‐5p inhibits tumour growth in nude mice by suppressing the EZH2/PI3K/AKT axis

At last, whether BMSC‐exo‐miR‐30b‐5p affects tumour growth in nude mice by regulating the EZH2/PI3K/AKT signalling axis was verified in in vivo experiments.

**FIGURE 4:**
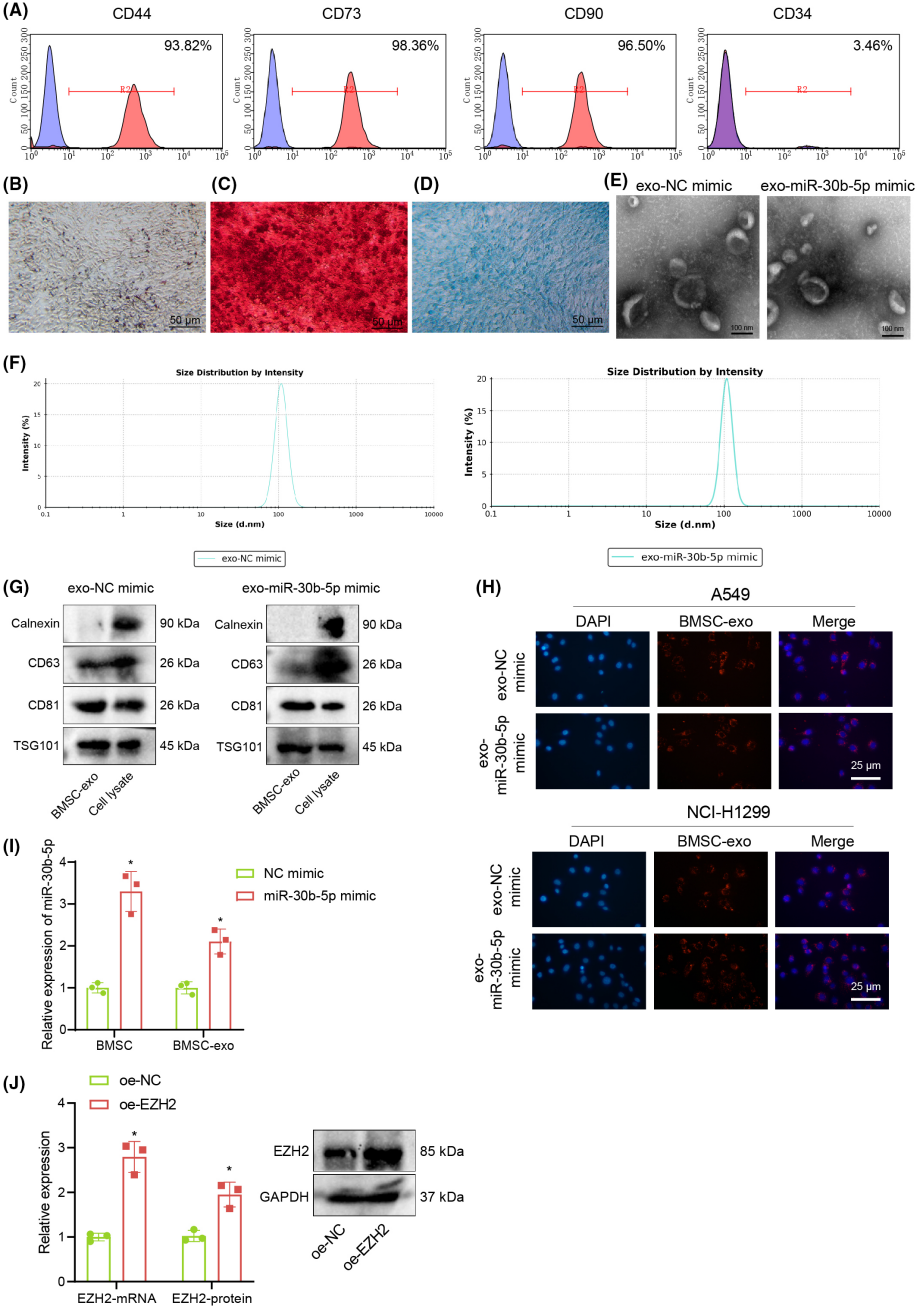
Identification of BMSC and BMSC‐exo. (A) Levels of BMSC surface markers (CD44, CD73, CD90 and CD34) identified by flow cytometry. (B) Distribution of lipid droplets in BMSCs cytoplasm after lipogenic induction in differentiation medium detected by Oil red O staining. (C) After 14 days of osteogenesis induction of BMSC, calcified nodules in cytoplasm observed by alizarin red staining. (D) Two weeks after chondrogenic induction of BMSC, chondrogenic ability of BMSCs detected by alcian blue staining. (E) Morphology of BMSC‐Exo observed under the TEM. (F) The size distribution of BMSC‐Exo detected by NTA. (G) Expression of Exo specific marker protein in BMSC‐exo and cell lysate detected by Western blot analysis. (H) Uptake of Exo by A549 or NCI‐H1299 cells detected by Immunofluorescence (PKH26‐labelled exo is red). (I) miR‐30b‐5p expression in BMSCs transduced with miR‐30b‐5p mimic and their derived Exo measured by RT‐qPCR. (J) Expression of EZH2 in A549 cells measured by RT‐qPCR and Western blot. *p < 0.05.

Flow cytometry was adopted to detect the expression of BMSC surface markers (CD44, CD73, CD90, CD34), which indicated that CD44 (93.82%), CD73 (98.36%) and CD90 (96.50%) were positive, while CD34 (3.46%) was negative (Figure 4A). BMSC adipogenic induction experiments showed that after about 2 weeks of BMSC adipogenic differentiation, lipid droplets were formed within the cytoplasm, and a large number of oil droplet vacuoles were observed by oil red O staining (Figure 4B). Moreover, after 14 days of osteogenic differentiation, numerous brown calcium nodules were observed in the cytoplasm by alzarin red staining (Figure 4C). Two weeks after BMSC chondrogenic induction, blue cytoplasm was observed with Alcian blue staining (Figure 4D). The above results indicated that the cells we cultured were BMSCs.

Next, miR‐30b‐5p was overexpressed in BMSCs, from which exosomes were extracted to detect the miR‐30b‐5p expression in BMSC and Exo. TEM and NTA showed that the isolated exosomes were the heterogeneous round or elliptical vesicles with a diameter of between 60 and 260 nm. Moreover, Western blot analysis displayed that CD63, CD81 and TSG101 were positively expressed, but Calnexin was not expressed (Figure 4E–G). The cell uptake assay suggested that after co‐incubation of PKH26‐labelled BMSCs‐exosomes and A549 cells or NCI‐H1299 cells, a large number of red fluorescent‐labelled BMSC exosomes were internalized by A549 cells or NCI‐H1299 cells under a confocal microscope (Figure 4H). RT‐qPCR exhibited that miR‐30b‐5p expression in BMSCs transduced with miR‐30b‐5p mimic and its secreted exosomes were higher than that in BMSCs transduced with NC mimic (Figure 4I). Moreover, RT‐qPCR and Western blot analysis displayed that mRNA and protein levels of EZH2 were elevated in A549 cells transduced with oe‐EZH2 (Figure 4J).

**FIGURE 5:**
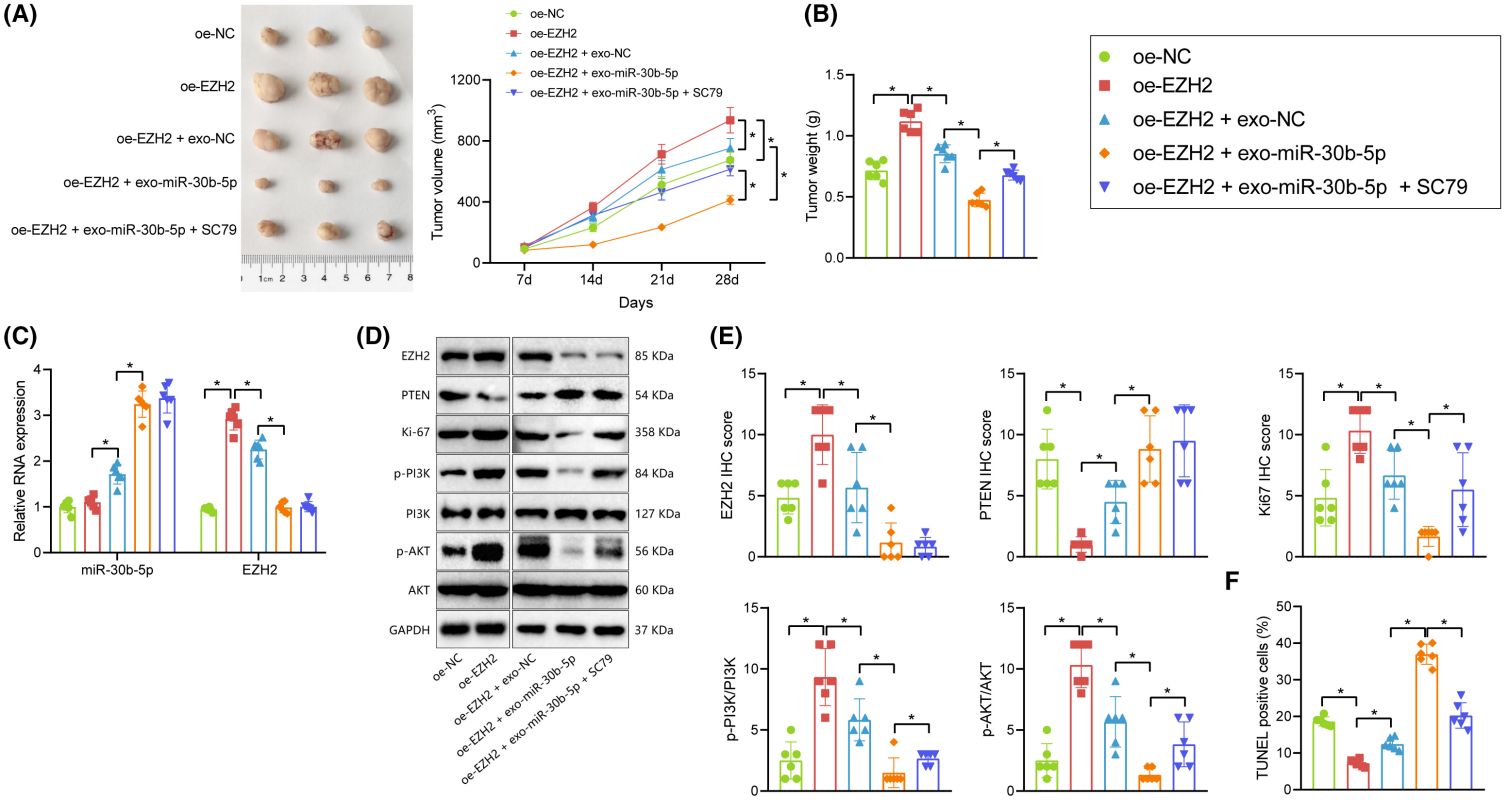
BMSC‐exo‐miR‐30b‐5p suppresses NSCLC growth in vivo via EZH2/PI3K/AKT axis. Nude mice were injected with oe‐EZH2, oe‐EZH2 + exo‐NC, EZH2 + exo‐miR‐30b‐5p or oe‐EZH2 + exo‐miR‐30b‐5p + SC79 (*n* = 6). (A) Tumour volume of nude mice. (B) Tumour weight of nude mice. (C) Expression of miR‐30b‐5p and EZH2 in tumour tissues of nude mice determined by RT‐qPCR. (D) Expression of EZH2, PTEN, p‐PI3K, p‐AKT and Ki‐67 in tumour tissues of nude mice determined by Western blot. (E) Expression of EZH2, PTEN, p‐PI3K, p‐AKT and Ki‐67 in tumour tissues of nude mice determined by IHC. (F) Apoptosis in tumour tissues of nude mice detected by TUNEL staining. *p < 0.05.

A549 cells overexpressing EZH2 were injected into the right axilla of nude mice for nude mice model establishment. The extracted exo‐NC and exo‐miR‐30b‐5p were injected around the tumour body, followed by the addition of the PI3K/AKT agonist SC79. It was found that compared with nude mice injected with A549 cells transduced with oe‐NC, tumour weight and volume were increased in nude mice injected with A549 cells transduced with oe‐EZH2, while compared with nude mice injected with A549 cells transduced with oe‐EZH2, tumour weight and volume were decreased in nude mice injected with A549 cells transduced with oe‐EZH2 and exo‐NC. Moreover, the tumour weight and volume of nude mice injected with A549 cells transduced with oe‐EZH2 and exo‐miR‐30b‐5p were lower than that of nude mice injected with A549 cells transduced with oe‐EZH2 and exo‐NC, while compared with nude mice injected with A549 cells transduced with oe‐EZH2 and exo‐miR‐30b‐5p, tumour weight and volume were elevated in nude mice injected with A549 cells transduced with oe‐EZH2, exo‐miR‐30b‐5p and SC79 (Figure 5A,B).

Tumour of nude mice was collected for RT‐qPCR, Western blot and IHC, which showed that expression of EZH2, p‐PI3K, p‐AKT and Ki‐67 was increased, but PTEN expression was reduced, while miR‐30b‐5p expression exhibited no difference in tumour tissues in the presence of oe‐EZH2. After further injection of exosomes, expression of EZH2, p‐PI3K, p‐AKT and Ki‐67 was decreased, while expression of PTEN and miR‐30b‐5p was elevated in tumour tissues in the presence of oe‐EZH2. Besides, expression of EZH2, p‐PI3K, p‐AKT and Ki‐67 was decreased, and expression of PTEN and miR‐30b‐5p was elevated in tumour tissues of nude mice injected with oe‐EZH2 + exo‐miR‐30b‐5p, but further addition of SC79 elevated expression of p‐PI3K, p‐AKT and Ki‐67, and exerted no alteration in expression of miR‐30b‐5p, EZH2 and PTEN (Figures 5C–E and S3A). TUNEL staining revealed that overexpression of EZH2 inhibited apoptosis, which was reversed by further treatment with exosomes. Moreover, exosomal miR‐30b‐5p combined with upregulated EZH2 induced apoptosis, while further treatment with SC79 suppressed apoptosis (Figures 5F and S3B).

The obtained data indicated that BMSC‐derived exosomal miR‐30b‐5p may promote the apoptosis of NSCLC cells and inhibit the tumour growth in nude mice by inhibiting EZH2/PI3K/AKT axis.

## 4 DISCUSSION

Bioengineered exosomes with desired cargoes and targeting specificity bear great responsibility in cancer therapy.^23^ Recently, a considerable literature has grown up around the theme of BMSC‐derived exosomes in suppressing tumour progression.^6^, ^24^, ^25^ In our current study, in silico analysis revealed that EZH2 may be an important downstream target gene of BMSC‐exo‐miRNAs and BMSC‐exo‐miRNAs may be involved in the progression of NSCLC by regulating the downstream target gene EZH2. Moreover, in vitro and in vivo experiments demonstrated that BMSC‐exo‐miR‐30b‐5p could inhibit EZH2 expression by activating the PI3K/AKT signalling pathway to suppress the progression of NSCLC. This study revealed the novel mechanism involved in NSCLC occurrence and development. The isolated BMSC‐exo served as a therapeutic strategy to affect the tumour growth and the study of downstream pathways also provided reference for clinical application.

Initially, it was found that a total of 86 differentially expressed BMSC‐exo‐miRNAs involved in the progression of NSCLC. Further GO and KEGG enrichment analysis for 86 differentially expressed BMSC‐exo‐miRNAs displayed that miRNAs were enriched in signal transduction, cell communication, TRAIL signalling pathway, VEGF and VEGFR signalling network, signal transduction, and cell communication. TRAIL pathway could suggest that BMSC‐exo‐miRNA possesses anti‐tumour activity through apoptosis induction,^26^ and VEGF/VEGFR may suggest the involvement of BMS‐exo‐miRNA in tumour angiogenesis.^27^, ^28^ Exosomes are a special class of carriers that deliver miRNAs into target cells to influence the development and progression of tumours.^29^, ^30^ A recent study has proved that miRNAs are aberrantly expressed in the development of NSCLC based on genomic sequencing and bioinformatics.^31^ Recent evidence suggests that exosomes‐encapsulated miRNAs could exert the anti‐tumour effects on NSCLC.^32^ It has been demonstrated that BMSC‐secreted exosomes shuffle miR‐193a to suppress cell proliferation, invasion and migration, as well as promote apoptosis to inhibit the NSCLC progression.^6^ Another study has also indicated that exosomal miR‐144 from BMSCs represses the development of NSCLC.^7^ Moreover, the current study has revealed that miR‐30b‐5p shuttled by BMSC‐derived exosomes is involved in the progression of NSCLC. Similarly, miR‐30b‐5p is downregulated in lung cancer, and miR‐30b‐5p could inhibit malignant phenotypes of cancer cells and tumorigenesis,^11^ which is consistent with our findings.

In addition, the current study has also indicated that BMSC‐derived exosomes carrying miR‐30b‐5p may participate in NSCLC progression by targeting EZH2. Multiple findings have exhibited that BMSC‐derived exosomes carrying miRNAs could suppress EZH2 expression to inhibit the tumour cell proliferation, migration and invasion, and induce its apoptosis, thereby restraining the development of tumours.^33^, ^34^ Evidence has been presented demonstrating that EZH2 expression is upregulated in NSCLC, which is related to the poor prognosis of NSCLC patients.^15^, ^35^ Numerous studies have suggested that that overexpression of EZH2 could promote the development, growth and metastasis of NSCLC, accompanied by the poor survival.^36^, ^37^ The obtained data elucidated that miR‐30b‐5p could target and repress EZH2 expression in NSCLC, indicating that miR‐30b‐5p may participate in NSCLC progression by targeting EZH2 in vitro.

Furthermore, in vivo experiments confirmed that BMSC‐secreted exosomes‐loaded miR‐30b‐5p suppressed EZH2/PI3K/AKT axis to enhance apoptosis and inhibit tumour growth in nude mice. It is known that miR‐30b‐5p suppresses PI3K/AKT signalling pathway to restrain the occurrence and progression of oesophageal squamous cell carcinoma.^38^ A previous study has demonstrated that EZH2 can promote H3K27me3 modification of PTEN promoter region, which inhibits PTEN expression and activates PI3K/AKT pathway to promote the proliferation and metastasis of NSCLC cells.^16^ As a phosphatase, the tumour suppressor protein PTEN can dephosphorylate Akt and reduce its activation, and can block all downstream signalling events regulated by Akt, and is a negative regulator of PI3K.^39^, ^40^ Another study has reported that EZH2 can upregulate PI3K/AKT signalling pathway to aggravate chronic lymphocytic leukaemia.^41^ PI3K/AKT signalling pathway, as one of the most significant intracellular pathways, affects cell survival and growth.^42^ The roles of PI3K/AKT signalling pathway in the development of NSCLC have also been validated.^43^ A recent study has also verified that PI3K/AKT signalling pathway is capable of inducing the tumorigenesis and the progression of NSCLC.^44^, ^45^ To the best of our knowledge, our results suggested that the miR‐30b‐5p shuttled by BMSC‐derived exosomes could inhibit the progression of NSCLC by targeting EZH2 via suppression of PI3K/AKT signalling pathway.

## 5 CONCLUSIONS

**FIGURE 6:**
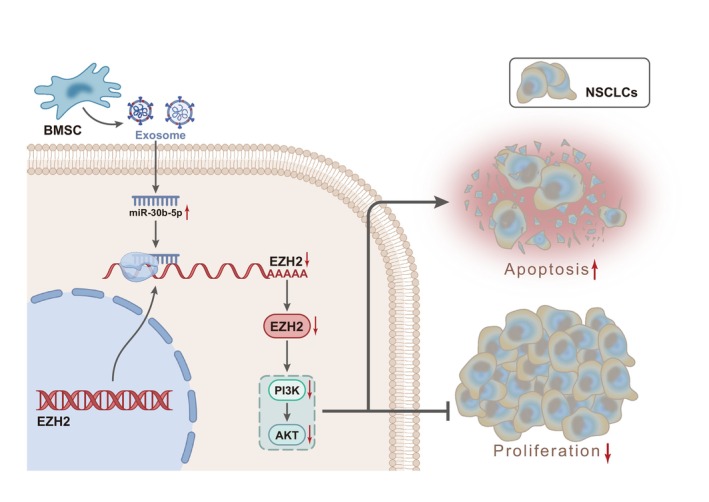
Molecular mechanisms of miR‐30b‐5p shuttled by BMSC‐derived exosomes in NSCLC mice. miR‐30b‐5p shuttled by BMSC‐derived exosomes suppressed NSCLC through inhibition of EZH2/PI3K/AKT axis.

In conclusion, it can be concluded that BMSC‐derived exosomes‐encapsulated miR‐30b‐5p could specifically inhibit EZH2 expression and PI3K/AKT signalling pathway activation to induce NSCLC cell apoptosis, thereby ultimately preventing NSCLC progression (Figure 6). This study reveals a new mechanism for the occurrence and development of NSCLC. The exosomes derived from BMSCs, as a novel therapeutic strategy, could affect tumour growth, and its downstream pathways provide a reference for clinical applications. In terms of mechanism, miR‐30b‐5p could inhibit EZH2 expression and inactivate PI3K/AKT signalling pathway. However, whether EZH2 regulates other genes to affect the expression of PTEN, PI3K/AKT pathway and Ki‐67 has not been deeply investigated in the current study. In addition, except for miR‐30b‐5p, BMSC‐derived Exo may contain other miRNAs that may have an impact on the progression of NSCLC, which will be further discussed in our future studies.

## AUTHOR CONTRIBUTIONS

**Tong Wu:** Conceptualization (equal); data curation (equal); formal analysis (equal); investigation (equal); methodology (equal); writing – original draft (equal); writing – review and editing (equal). **Qi Tian:** Conceptualization (equal); data curation (equal); formal analysis (equal); investigation (equal); methodology (equal); writing – original draft (equal); writing – review and editing (equal). **Ruiji Liu:** Data curation (equal); formal analysis (equal); resources (equal); software (equal). **Ke Xu:** Data curation (equal); formal analysis (equal); software (equal). **Shanshan Shi:** Investigation (equal); supervision (equal); validation (equal). **Xiudi Zhang:** Data curation (equal); validation (equal); visualization (equal); writing – review and editing (equal). **Liming Gao:** Investigation (equal); validation (equal); visualization (equal); writing – review and editing (equal). **Xiaobo Yin:** Supervision (equal); validation (equal); visualization (equal); writing – review and editing (equal). **Shufeng Xu:** Conceptualization (equal); funding acquisition (equal); project administration (equal); writing – original draft (equal); writing – review and editing (equal). **Ping Wang:** Conceptualization (equal); funding acquisition (equal); project administration (equal); writing – original draft (equal); writing – review and editing (equal).

## FUNDING INFORMATION

This work is supported by Natural Science Foundation of Hebei Province (H2021107002).

## CONFLICT OF INTEREST STATEMENT

The authors declare that they have no competing interests.

## Supporting information


Figure S1.

Figure S2.

Figure S3.

Table S1.

Table S2.

Table S3.

Table S4.

Table S5.

## Data Availability

The data underlying this article will be shared on reasonable request to the corresponding author.
