# Supplementary figures and images for: Inhibitory role of bone marrow mesenchymal stem cells‐derived exosome in non‐small‐cell lung cancer: microRNA‐30b‐5p, EZH2 and PI3K/AKT pathway
Source: J Cell Mol Med. 2023 Sep 12;27(22):3526–38. doi: 10.1111/jcmm.17933 (PMC10660609; doi:10.1111/jcmm.17933)

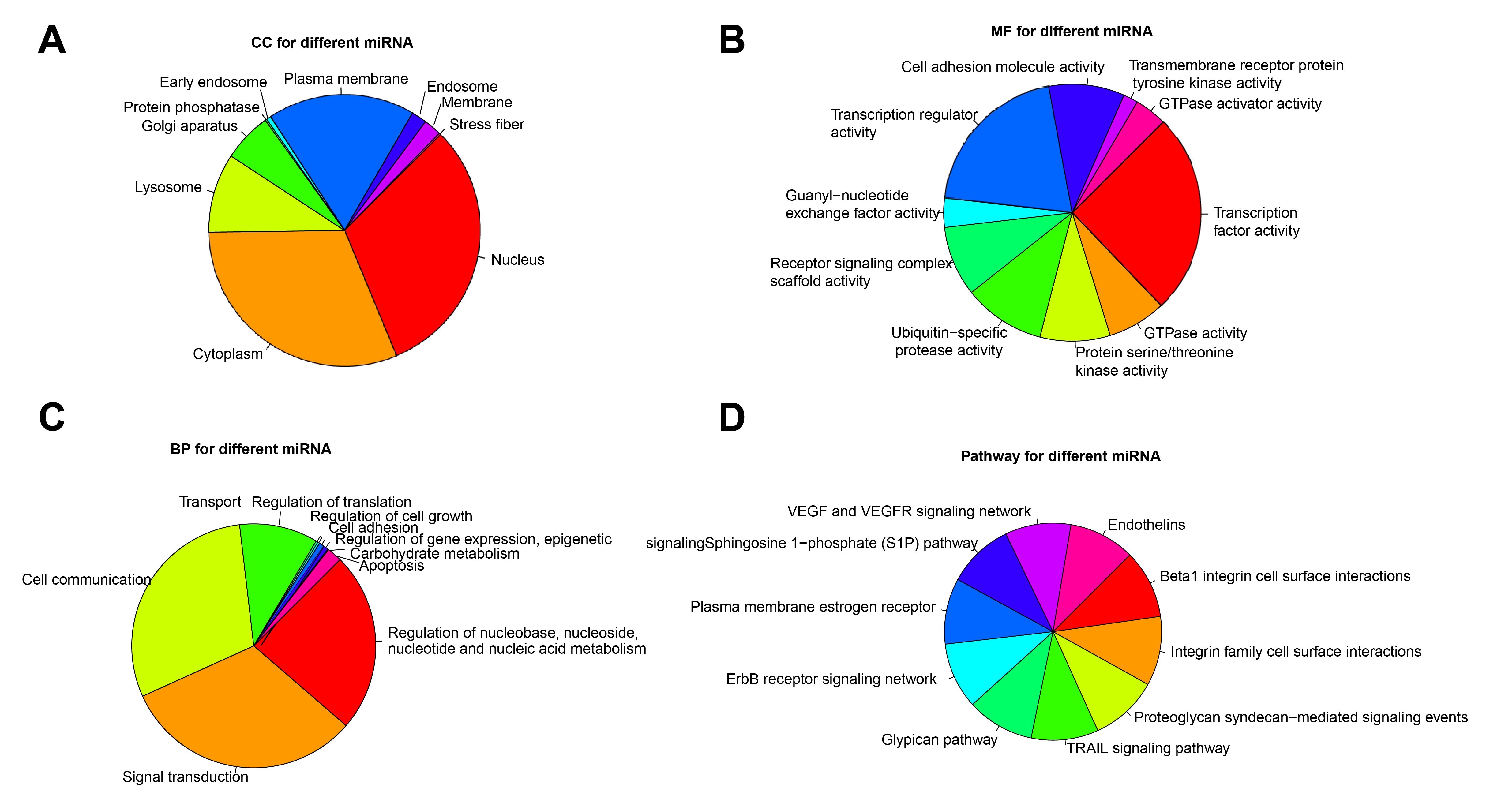

Supplement: Supplementary file 1 — Figure S1. [file JCMM-27-3526-s002.jpg]

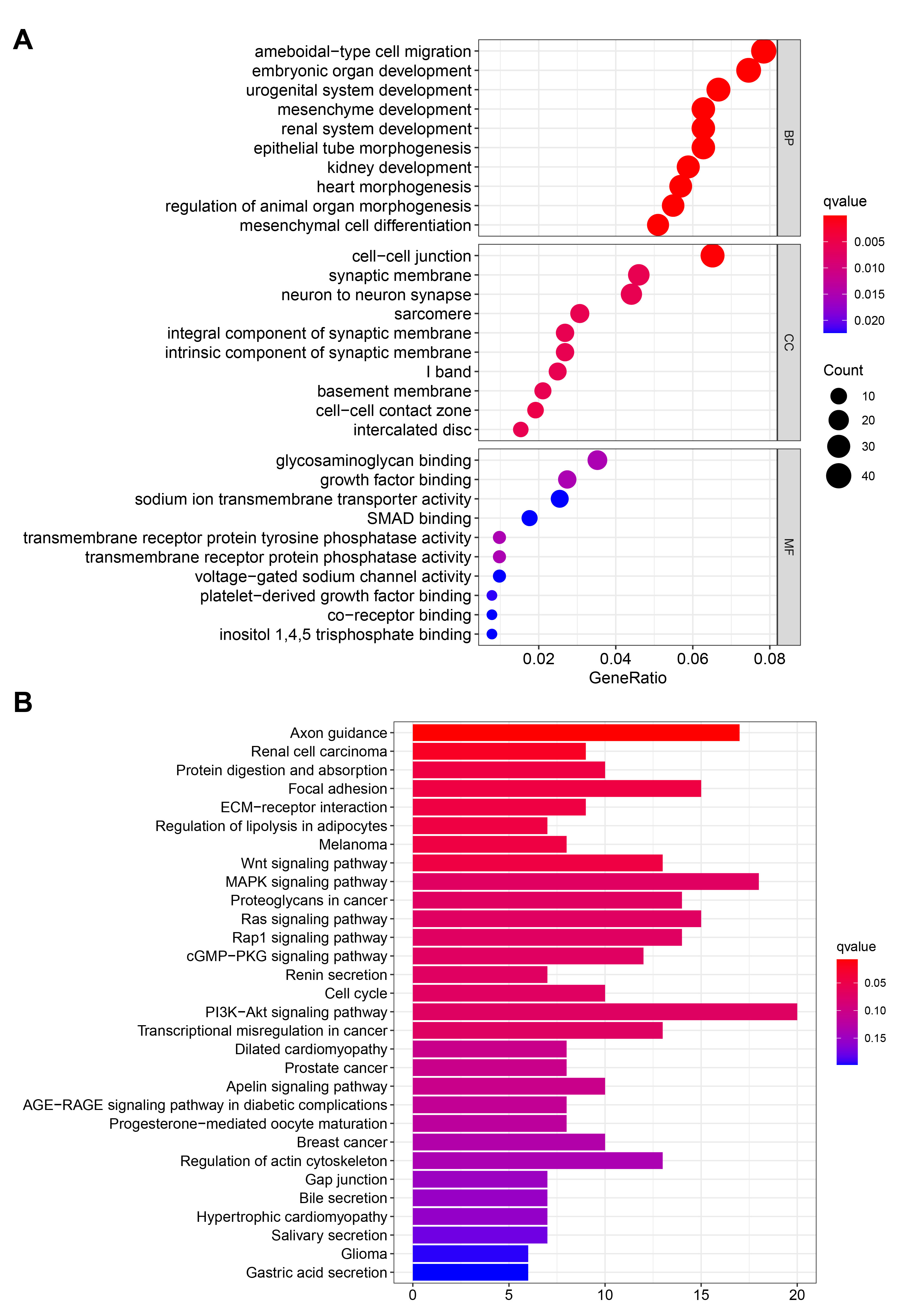

Supplement: Supplementary file 2 — Figure S2. [file JCMM-27-3526-s007.jpg]

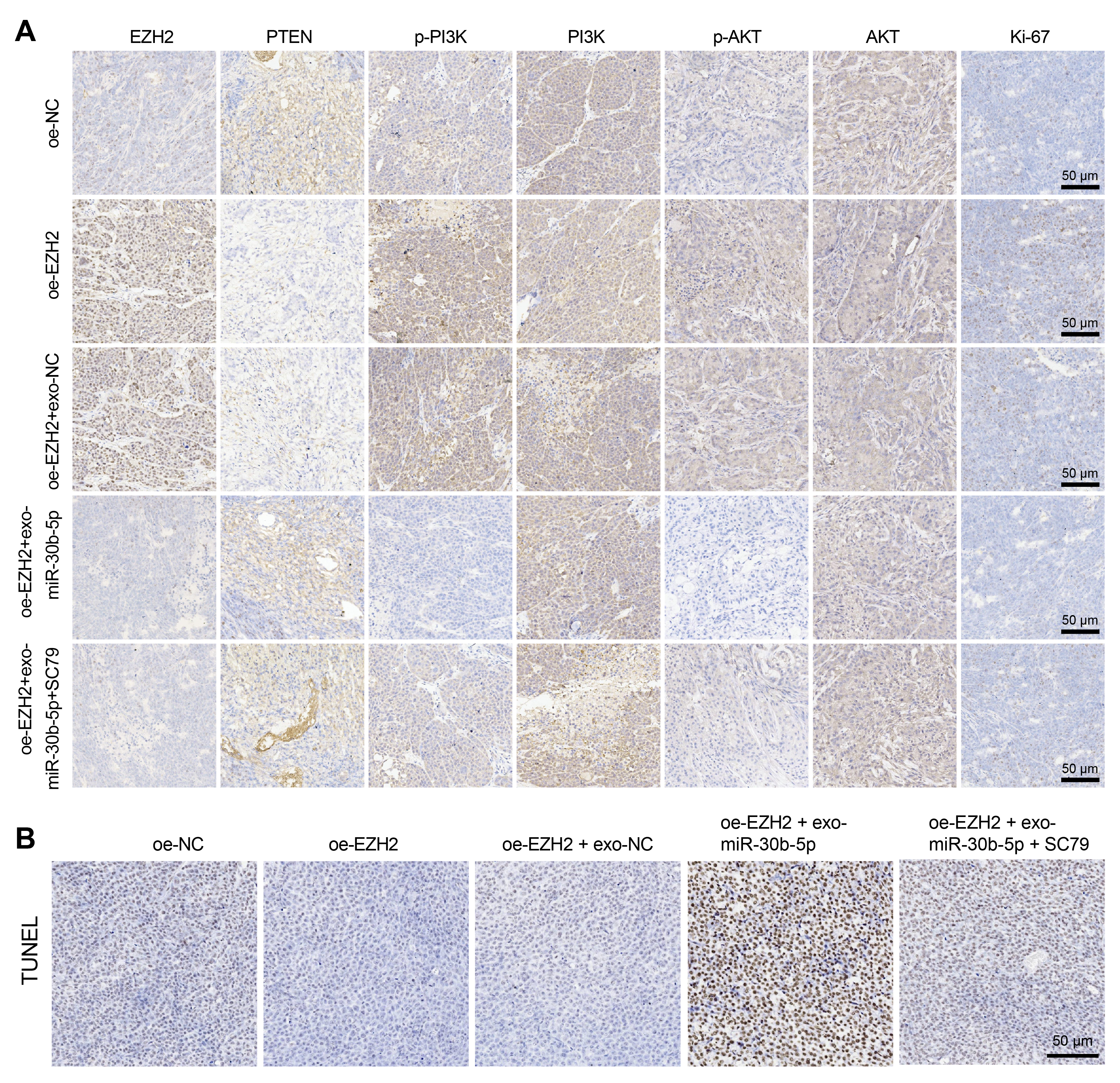

Supplement: Supplementary file 3 — Figure S3. [file JCMM-27-3526-s001.jpg]
