# Supplementary material for: Inhibitory role of bone marrow mesenchymal stem cells‐derived exosome in non‐small‐cell lung cancer: microRNA‐30b‐5p, EZH2 and PI3K/AKT pathway
Source: J Cell Mol Med. 2023 Sep 12;27(22):3526–38. doi: 10.1111/jcmm.17933 (PMC10660609; doi:10.1111/jcmm.17933)
Supplement: Supplementary file 4 — Table S1. [file JCMM-27-3526-s006.docx]

**Table S1** Primer sequences of RT-qPCR

| Gene | Primer sequences (5’-3’) |
| --- | --- |
| miR-30b-5p | Forward: TGTAAACATCCTACACTCAGCT |
|  | Reverse: Universal reverse primer |
| U6 | Forward: CTCGCTTCGGCAGCACA |
|  | Reverse: Universal reverse primer |
| EZH2 | Forward: GGACTCAGAAGGCAGTGGAG |
|  | Reverse: CTTGAGCTGTCTCAGTCGCA |
| GAPDH | Forward: GACAGTCAGCCGCATCTTCT |
|  | Reverse: GCGCCCAATACGACCAAATC |

Note: RT-qPCR, reverse transcription quantitative polymerase chain reaction; miR-30b-5p, microRNA-30b-5p; EZH2, enhancer of zeste homolog 2; GAPDH, glyceraldehyde-3-phosphate dehydrogenase.
